# Antigen Localization Influences the Magnitude and Kinetics of Endogenous Adaptive Immune Response to Recombinant Salmonella Vaccines

**DOI:** 10.1128/IAI.00593-17

**Published:** 2017-11-17

**Authors:** Yanina R. Sevastsyanovich, David R. Withers, Claire L. Marriott, Faye C. Morris, Timothy J. Wells, Douglas F. Browning, Irene Beriotto, Ewan Ross, Hossam Omar Ali, Catherine A. Wardius, Adam F. Cunningham, Ian R. Henderson, Amanda E. Rossiter

**Affiliations:** a Institute of Microbiology and Infection, College of Medical and Dental Sciences, University of Birmingham, Birmingham, United Kingdom; b Institute of Immunology and Immunotherapy, College of Medical and Dental Sciences, University of Birmingham, Birmingham, United Kingdom; University of California, Davis

**Keywords:** Salmonella, autotransporter proteins, vaccines

## Abstract

The use of recombinant attenuated Salmonella vaccine (RASV) strains is a promising strategy for presenting heterologous antigens to the mammalian immune system to induce both cellular and humoral immune responses. However, studies on RASV development differ on where heterologous antigens are expressed and localized within the bacterium, and it is unclear how antigen localization modulates the immune response. Previously, we exploited the plasmid-encoded toxin (Pet) autotransporter system for accumulation of heterologous antigens in cell culture supernatant. In the present study, this Pet system was used to express early secretory antigen 6 (ESAT-6), an immunodominant and diagnostic antigen from Mycobacterium tuberculosis, in Salmonella enterica serovar Typhimurium strain SL3261. Three strains were generated, whereby ESAT-6 was expressed as a cytoplasmic (SL3261/cyto), surface-bound (SL3261/surf), or secreted (SL3261/sec) antigen. Using these RASVs, the relationship between antigen localization and immunogenicity in infected C57BL/6 mice was systematically examined. Using purified antigen and specific tetramers, we showed that mice infected with the SL3261/surf or SL3261/sec strain generated large numbers of Th1 CD4^+^ ESAT-6^+^ splenic T cells compared to those of mice infected with SL3261/cyto. While all mice showed ESAT-6-specific antibody responses when infected with SL3261/surf or SL3261/sec, peak total serum IgG antibody titers were reached more rapidly in mice that received SL3261/sec. Thus, how antigen is localized after production within bacteria has a more marked effect on the antibody response than on the CD4^+^ T cell response, which might influence the chosen strategy to localize recombinant antigen in RASVs.

## INTRODUCTION

Vaccines based on attenuated bacterial strains can generate protective immunity against disease caused by the parent strain (1–3). Attenuated strains of Salmonella spp. can be modified to express heterologous antigens from a range of viral, bacterial, protozoan, and fungal agents and, as such, have been named recombinant attenuated Salmonella vaccine (RASV) strains (4–10). The capacity of RASVs to elicit protective immune responses is heavily dependent upon the subcellular localization of antigen expression. For example, many systems have been developed to overexpress heterologous antigens within the cytoplasm (11). However, these systems often show poor immunogenicity in mice, and their application in vaccine development has been questioned (12). In light of this, different bacterial secretion systems (e.g., types 1, 3, and 5) have been exploited to target recombinant antigens to the bacterial cell surface or extracellular milieu (13–20).

The type 5 autotransporter (AT) secretion system represents the most simplistic molecular machinery for protein secretion in Gram-negative bacteria (21–26). Multiple AT platforms have been exploited to present antigens on the bacterial cell surface, including fusions with Ag43, AIDA-I, and Hbp from Escherichia spp. and MisL from Salmonella spp., and this approach has been termed autodisplay (27–30). Many studies have reported antigen-specific cellular and humoral responses to heterologous antigens presented via autodisplay and, in some cases, protection against challenge infection (17, 30–32). Although autodisplay has become a system of choice for recombinant protein expression for various applications, there are limited studies comparing the effects of secreted versus cell surface-bound antigen on host immune responses (33). Nevertheless, some studies have suggested that protrusion of the target antigen away from bacterial cell surface structures may facilitate protective immunity (34). This implies that the mode of antigen presentation to the immune system, rather than intrinsic properties of the antigen and/or expression levels, can determine immunogenicity. Thus, there is a need to understand how the cellular localization of an antigen influences the immune response induced by RASVs.

In this study, we utilized the type 5 autotransporter plasmid-encoded toxin (Pet) (18), which can be modified to support the accumulation of secreted or surface-bound recombinant antigen. Thus, this platform presents a unique opportunity to directly compare the influences of differentially localized antigens on host immune responses. Using Salmonella enterica serovar Typhimurium strain SL3261, we exploited Pet to deliver cytoplasmic, secreted, and surface-bound forms of a model antigen to the immune system. Early secretory antigen 6 (ESAT-6) of Mycobacterium tuberculosis was chosen as the model antigen because it is a nonnative protein which induces specific cells that can be detected *ex vivo*. *S*. Typhimurium SL3261 was chosen for delivery because it is an attenuated strain that is able to cause a sustained yet limited infection of mice and disseminates to multiple organs, such as the spleen and liver (35). Mice infected with such attenuated strains of Salmonella spp. resolve the infection over a period of 5 to 6 weeks through the induction of CD4^+^ Th1 cells and IgG antibody (35). The defined kinetics of this Salmonella infection allows antigen-specific T cell and antibody responses to be monitored. Our studies show that cell surface-bound or secreted antigen drives a significantly larger proportion of ESAT-6-specific T cells than that seen with cytoplasmic antigen. Furthermore, we show that the total ESAT-6-specific serum IgG antibody levels at 21 days postinfection are significantly higher when ESAT-6 is presented as a secreted antigen. Our data present a detailed comparison of AT-mediated secretion versus surface presentation of a recombinant antigen in RASV-infected mice and provide novel insights into the nature of cellular and humoral immune responses against antigens localized to different subcellular compartments within the RASV strain.

## RESULTS

### Construction and characterization of RASVs expressing ESAT-6-Pet chimeric proteins.

**FIG 1:**
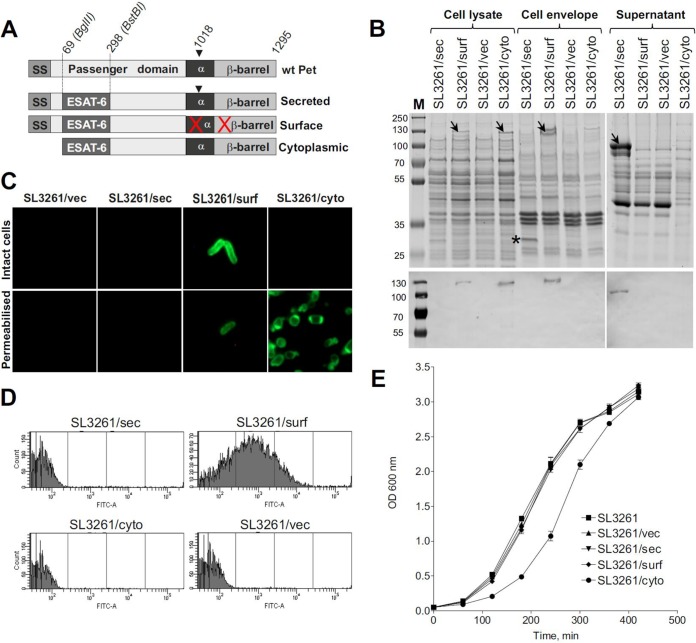
Construction and characterization of recombinant attenuated Salmonella vaccine (RASV) strains. (A) Schematic of Pet AT and the secreted, surface-bound, and cytoplasmic derivatives containing ESAT-6, cloned within the indicated BglII and BstBI restriction sites. The following Pet domains are shown: signal sequence (SS), passenger domain, α-helix (α), and β-barrel. The numbers above Pet denote amino acid positions. Amino acid position 1018 indicates where intrabarrel cleavage occurs to mediate secretion. The red crosses denote amino acid substitutions within the α-helix and β-barrel which enable surface localization of the Pet chimera (18). (B) SDS-PAGE (top) and Western blot (bottom) analyses of ESAT-6-Pet protein localization in cell lysate, envelope, and supernatant fractions prepared from the indicated RASV strains expressing vector only (SL3261/vec) or the cytoplasmic (SL3261/cyto), surface-bound (SL3261/surf), or secreted (SL3261/sec) ESAT-6-Pet fusion. The expressed protein fusions are indicated by arrows in the SDS-PAGE gel and were also detected by Western immunoblotting with anti-ESAT-6. Lane M, protein markers (kilodaltons). Note the additional 30-kDa band (marked with asterisk) appearing in the cell envelope fraction of SL3261/sec, which corresponds to the Pet β-barrel localized in the outer membrane. (C and D) Confirmation of subcellular localization of ESAT-6-Pet fusions in the indicated RASV strains by immunofluorescence (C) and flow cytometry (D) analyses. Protein fusions were detected using antibodies specific to ESAT-6 in conjunction with fluorescein isothiocyanate (FITC)-conjugated secondary antibodies. Nonpermeabilized cells were used for flow cytometry, while permeabilized cells were also used in the immunofluorescence analysis to allow intracellular labeling of the cytoplasmic ESAT-6-Pet fusion. (E) Growth kinetics of the untransformed SL3261 strain and the indicated RASV strains. Overnight cultures were subcultured into fresh LB broth and normalized to an OD_600_ of 0.05 before measurements were taken every hour for 7 h. Individual data points represent means ± SEM for three biological replicates.

Previously, we showed that the Pet autotransporter can be exploited to secrete a range of heterologous proteins into the culture medium in a natively folded and functional state (18). Moreover, a cleavage-deficient Pet variant, Pet*, can be used to assemble heterologous proteins on the bacterial cell surface. Here we generated three ESAT-6-Pet chimeric constructs, pettac-ESAT6-Pet, pettac-ESAT6-Pet*, and pettac-ESAT6-cyto, expressing secreted, surface-bound, and cytoplasmic ESAT-6-Pet proteins, respectively (Fig. 1A and Table 1). These fusions were cloned into the pettac vector, a modified pET22b+ plasmid with the T7 promoter exchanged for a *tac* promoter, allowing constitutive gene expression (36). This vector was tested previously for its stability and validity in antigen expression for immunization studies with Salmonella species (36, 37). The recombinant plasmids, including a vector-only control, were transformed into a well-characterized RASV strain (Salmonella enterica serovar Typhimurium SL3261 *aroA*) (10). From here on, the SL3261 strains expressing ESAT-6 in surface-bound, secreted, and cytoplasmic forms are referred to as SL3261/surf, SL3261/sec, and SL3261/cyto, respectively. All ESAT-6-expressing RASV strains were tested for ESAT-6-Pet expression. Whole-cell lysates and outer membrane and secreted protein fractions were prepared from bacterial cultures harvested at the logarithmic phase of growth. Figure 1 demonstrates that ESAT-6-Pet was localized to the expected subcellular compartments as examined by Western immunoblotting (Fig. 1B), indirect immunofluorescence assay (Fig. 1C), and flow cytometry analysis (Fig. 1D). Furthermore, using whole-cell lysates, cell envelope and supernatant fractions, and densitometric analysis of Western blots, we confirmed that ESAT-6 levels in the cytoplasmic, supernatant, and membrane fractions were comparable between the strains at the exponential phase of growth, with only the SL3261/sec strain displaying a slight increase of ESAT-6 production (see Fig. S1 in the supplemental material). All strains grew with similar kinetics during the logarithmic phase, although SL3261/cyto had a slightly extended lag phase (Fig. 1E). Thus, we generated RASV strains that are able to localize ESAT-6-Pet expression to different cellular compartments and that show comparable growth rates and antigen expression levels.

**TABLE 1:** Bacterial strains and plasmids used in this study

Bacterial strain or plasmid	Description	Source or reference
Strains		
E. coli NEB 5-alpha	*fhuA2* Δ*(argF-lacZ)U169 phoA glnV44* ϕ*80* Δ*(lacZ)M15 gyrA96 recA1 relA1 endA1 thi-1 hsdR17*	NEB
E. coli BL21*(DE3)	F^−^ *ompT gal dcm lon hsdS*_B_(r_B_^−^ m_B_^−^) *rne131* λ(DE3 [*lacI lacUV5-T7 gene 1 ind-1 sam-7 nin-5*])	Novagen
Salmonella enterica Typhimurium SL3261	Salmonella enterica serovar Typhimurium SL1344 derivative containing an *aroA* deletion	10
Plasmids		
pASK-ESAT6-Pet-BB	pASK-Pet with *esxA* (ESAT-6) insertion between BglII and BstBI sites of wild-type *pet* gene	18
pASK-ESAT6-Pet*	pASK-Pet with *esxA* (ESAT-6) insertion between BglII and BstBI sites of cleavage-deficient *pet* gene	18
pASK-His_6_-Pet	pASK vector expressing His_6_-tagged Pet	18
pettacOVA	Modified pET22b+ with ovalbumin gene cloned between NdeI and XhoI sites; Amp^r^	36
pettac	pettacOVA derivative generated by OVA excision and vector religation	This study
pettac-ESAT6-Pet	pettac expressing secreted ESAT-6-Pet	This study
pettac-ESAT6-Pet*	pettac expressing surface-bound ESAT-6-Pet	This study
pettac-ESAT6-Pet-cyto	pettac expressing cytoplasmic ESAT-6-Pet	This study
pASK-His_6_-ESAT6-Pet-BP	pASK-His_6_-Pet derivative containing *esxA* (ESAT-6) insertion between BglII and PstI sites of *pet*	This study
pET-His_6_-ESAT6-Pet-BP	pET22b expressing His_6_-ESAT6-Pet fusion	This study

### RASV strains expressing surface-bound and secreted ESAT-6 elicit significant ESAT-6-specific T cell responses.

**FIG 2:**
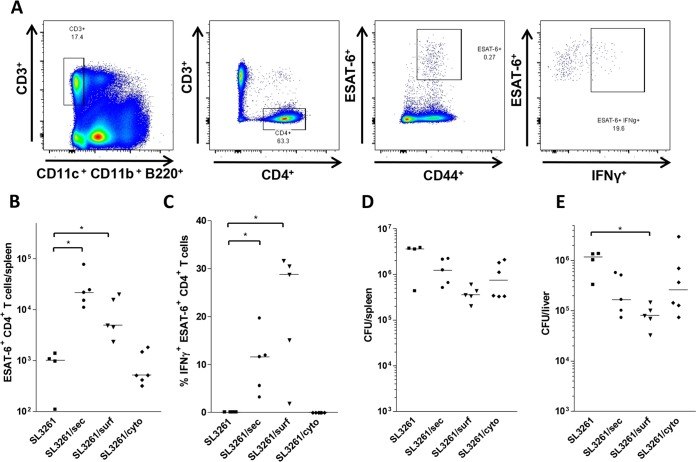
SL3261/sec and SL3261/surf elicit ESAT-6-specific T cell responses in immunized mice. C57BL/6 mice were infected with untransformed Salmonella SL3261 and the SL3261/sec, SL3261/surf, and SL3261/cyto RASV strains, expressing secreted, surface, and cytoplasmic ESAT-6, respectively. At 7 days postinfection, mice were injected i.v. with an ESAT-6 peptide for *in vivo* restimulation of ESAT-6-specific T cell populations. Splenic ESAT-6^+^ CD4^+^ T cells were detected by staining with an ESAT-6 MHC II tetramer and anti-IFN-γ to assess cytokine production. (A) Representative flow cytometry plots of splenic lymphocyte populations from an RASV-immunized mouse, showing the gating sequence to identify ESAT-6^+^ IFN-γ^+^ CD4^+^ T cells. The labels below the flow cytometry plots indicate the phenotypes of the cells present within the gated regions. (B) Numbers of ESAT-6-specific CD4^+^ T cells in spleens of mice immunized with the indicated RASV strains, detected using an ESAT-6 MHC II tetramer. (C) Proportions of cells producing IFN-γ in the responding ESAT-6^+^ CD4^+^ T cell populations shown in panel B. (D and E) Numbers of CFU recovered from mouse spleens (D) and livers (E) at 7 days postinfection. Data from two experiments are shown, with a combined cohort size of 4 to 6 mice. *P* values were calculated using the Mann-Whitney *t* test (*, *P* ≤ 0.05; **, *P* ≤ 0.01; ***, *P* ≤ 0.001). A median bar is shown for each group.

We next sought to investigate the endogenous T cell response to differentially presented antigen. To do this, we analyzed the number of ESAT-6-specific CD4^+^ T cells and their capacity to produce the classical Th1-associated cytokine, gamma interferon (IFN-γ), using mouse-specific tetramers and intracellular cytokine staining. Wild-type mice were infected intraperitoneally (i.p.) with 10^5^ CFU of SL3261/surf, SL3261/sec, SL3261/cyto, or wild-type SL3261. At 7 days postinfection, mice were injected intravenously (i.v.) with an ESAT-6 peptide to restimulate ESAT-6-specific T cells. Four hours later, splenocytes were harvested, and CD4^+^ T cells were stained for specificity for ESAT-6 and IFN-γ expression (a representative gating strategy is shown in Fig. 2A). The number of CD4^+^ ESAT-6^+^ specific T cells in the spleen increased for mice infected with SL3261/surf or SL3261/sec compared to that for mice infected with SL3261/cyto or the SL3261 control strain. Furthermore, after infection with SL3261/surf or SL3261/sec, 10 to 30% of ESAT-6-specific T cells produced IFN-γ, indicating differentiation to a Th1 phenotype. The proportion of T cells producing IFN-γ tended to be higher in the SL3261/surf-infected mice than in the SL3261/sec-infected mice (Fig. 2C), although the difference was not significant. The failure to observe ESAT-6^+^ T cells after immunization with SL3261/cyto was not due to defects in bacterial colonization between the ESAT-6 strains, as bacteria persisted in similar numbers for all ESAT-6-producing strains (Fig. 2D and E). Importantly, there were no significant differences in plasmid stability in bacteria recovered from mouse spleens and livers postimmunization, as assessed by colony replica plating on nutrient agar with and without carbenicillin. All constructs showed between ∼80 and 100% plasmid retention (Table S1). These results indicate that SL3261/surf and SL3261/sec, but not SL3261/cyto, are capable of inducing an endogenous, antigen-specific CD4^+^ T cell response.

### Secreted and surface-bound ESAT-6 antigens drive a specific and sustained T cell response in infected mice.

**FIG 3:**
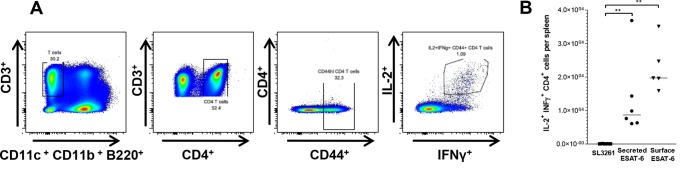
Characterization of T cell immune responses to ESAT-6-expressing RASV strains at 42 days postinfection. C57BL/6 mice were infected with Salmonella SL3261 strains expressing secreted or surface-bound ESAT-6 or with the untransformed SL3261 control. At 42 days postimmunization, ESAT-6-specific T cells were restimulated with an ESAT-6 peptide. Functional CD4^+^ T cells were identified by staining for the intracellular cytokines IFN-γ and IL-2. (A) Representative flow cytometry plots of stained splenocytes from an RASV-immunized mouse, showing the gating sequence to identify IFN-γ^+^ IL-2^+^ CD4^+^ T cells. (B) Numbers of IFN-γ^+^ IL-2^+^ CD4^+^ T cells in spleens of mice infected with ESAT-6-expressing RASV strains or a negative control (SL3261). Mouse cohorts included 5 or 6 animals. The data shown are representative of two independent experiments. *P* values were calculated using the Mann-Whitney *t* test (*, *P* ≤ 0.05; **, *P* ≤ 0.01; ***, *P* ≤ 0.001). A median bar is shown for each group.

Having shown the induction of ESAT-6-specific T cell responses in early infection, we next sought to investigate whether these T cell responses to secreted and surface-bound ESAT-6 were sustained over the course of infection. To do this, mice were infected with the indicated SL3261 strains, and at 42 days postinfection, the cytokine-producing (IL-2^+^ IFN-γ^+^) T cell response was examined (the gating strategy is shown in Fig. 3A). Immunization with ESAT-6-expressing RASVs produced a significant proportion of CD4^+^ IL-2^+^ IFN-γ^+^ T cells. These T cells were readily detectable in the spleens of both groups that were immunized with ESAT-6-expressing strains, although nearly twice as many were detected in spleens of mice that received SL3261/surf (Fig. 3B). This difference was not due to a difference between the levels of ESAT-6 expressed by the immunized strains, as levels of ESAT-6 production were similar for all strains recovered from infected mice at day 42 (data not shown). Moreover, there was no correlation between numbers of responsive splenic T cells and numbers of bacteria per spleen (data not shown). Thus, both SL3261/surf and SL3261/sec can induce long-term CD4^+^ Th1 cell responses.

### Secreted antigen changes the dynamics of the ESAT-6-specific antibody response during the course of Salmonella infection.

**FIG 4:**
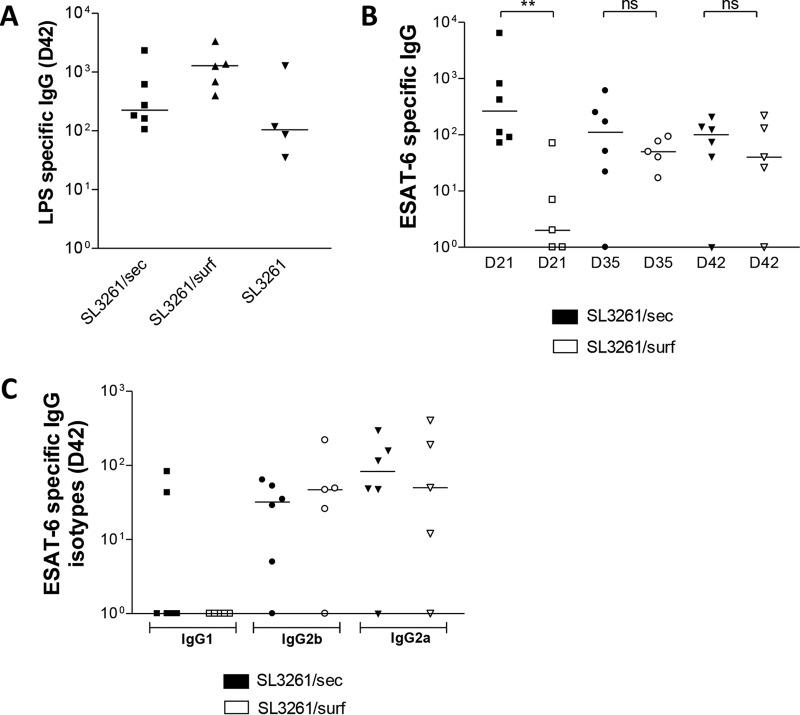
ESAT-6-specific antibody responses in RASV-immunized mice. (A and B) Salmonella LPS-specific (A) and ESAT-6-specific (B) IgG titers in mouse blood samples taken 21 (D21), 35 (D35), and 42 (D42) days after infection with the indicated RASV strains. (C) Analysis of ESAT-6-specific IgG isotypes in blood samples taken at 42 days postimmunization. Antibody titers were determined using the specified threshold OD method. A median bar is shown for each group. The data are representative of two independent experiments. Each mouse cohort included 5 or 6 mice. *P* values were calculated using the Mann-Whitney *t* test (*, *P* ≤ 0.05; **, *P* ≤ 0.01; ***, *P* ≤ 0.001; ns, not significant). A median bar is shown for each group.

Given that endogenous T cells did not respond to cytoplasmic ESAT-6 (SL3261/cyto), this variant was excluded from subsequent analyses. To investigate the antibody responses induced by the two ESAT-6-expressing strains, mice were infected i.p. for periods of up to 42 days, and blood samples were taken at days 21, 35, and 42 to measure ESAT-6 and lipopolysaccharide (LPS) IgG antibody titers. Marked antibody responses to the immunodominant Salmonella antigen LPS were detected at all time points examined for mice infected with ESAT-6-expressing strains (representative data for day 42 are shown in Fig. 4A). ESAT-6-specific total IgG antibody titers were 100-fold higher in mice infected with SL3261/sec than in those infected with SL3261/surf at 21 days postinfection, while similar IgG levels were detected between these strains at later time points (Fig. 4B). Assessment of IgG isotypes at day 42 postinfection showed that IgG2a and IgG2b, but not IgG1, were detected, with no difference in isotype pattern observed between SL3261/sec- and SL3261/surf-infected mice. These results indicate that mice were equally responsive to Salmonella infection, yet SL3261/sec induced peak anti-ESAT-6 IgG antibody titers more rapidly than SL3261/surf did.

## DISCUSSION

Multiple studies have detailed the use of RASVs to generate protective immunity against heterologous pathogens; however, the effect of antigen localization within RASVs remains unclear (4, 5, 7). Here we provide a systematic comparison of T and B cell responses to differentially presented ESAT-6, which can likely inform the design of RASVs in vaccine development. Multiple studies have described immunogenicity against target antigens presented for surface localization by autodisplay in Salmonella spp. (17, 31, 32). However, this is the first time that the AT system has been used to investigate immune responses to ESAT-6 *in vivo* by using tetramers to detect antigen-specific T cells in the context of RASV development. T cell responses to ESAT-6 are used clinically to identify latent tuberculosis infections, and this antigen contains multiple epitopes that are recognized by humans and mice. Additionally, Mollenkopf et al. developed a RASV that secreted ESAT-6 and showed that vaccination of mice with a single dose of Salmonella could lead to a reduction in the numbers of tubercle bacilli in the lungs after subsequent challenge with M. tuberculosis (38). Thus, this makes understanding the relationship between immune responses and antigen localization particularly relevant for translation to humans.

The differences observed in the T cell responses to this antigen are not likely to reflect differences in colonization by the different RASV strains used, as the strains grew well *in vitro*, readily expressed ESAT-6, were recoverable at similar levels *in vivo*, and retained their respective plasmids. Furthermore, the influence of the site of antigen expression is emphasized by the finding that SL3261/cyto showed similar levels of antigen production yet induced minimal T cell responses. Thus, any differences observed in the immunogenicity of ESAT-6 were more likely to be due to antigen localization than to differences in colonization.

In mice receiving SL3261/cyto, we were unable to detect ESAT-6-specific T cell responses. The differences in these results may lie in the accessibility of antigen to antigen-presenting cells. If so, then this suggests that there may be too few peptides derived from cytoplasm-restricted antigens presented via major histocompatibility complex class II (MHC II) to compete for rare endogenous T cell responses. Indeed, it may be significant that, for Salmonella spp., the majority of T cell epitopes identified originate from surface or secreted proteins (39, 40). Nevertheless, T cell responses to cytoplasmically localized ESAT-6 have been described previously (41). The reasons for the discrepancy between the study of Hall and colleagues (41) and the current study are unclear and may relate to the different approaches used to identify antigen-specific T cell responses.

In contrast to the absence of T cell responses to SL3261/cyto, all mice infected with SL3261/surf and SL3261/sec generated significant numbers of ESAT-6-specific T cells compared to those with SL3261 alone. Many of the ESAT-6-specific T cells had a Th1 phenotype, reflecting the Th1 response known to be induced in response to *S*. Typhimurium in the spleen at the examined time points (42, 43). Immunization with SL3261/surf tended to result in a greater specific Th1 response than that seen after immunization with SL3261/sec, a trend that was consistent at both 7 (Fig. 2C) and 42 (Fig. 3B) days postinfection. It is unclear why SL3261/sec should lead to a smaller number of Th1 cells, but this may be due to a stronger adjuvant effect of T cell receptor (TCR) ligands on the cell surface leading to their co-uptake by the same antigen-presenting cell. It is also important that our previous studies examining the endogenous Th1 response to Salmonella infection revealed that all IFN-γ^+^ CD4^+^ T cells expressed T-bet, a transcription factor important for the control of Salmonella, yet not all T-bet-expressing CD4^+^ T cells were IFN-γ^+^ (44). Thus, these RASV strains may drive the expansion of additional T cell subsets, and further work is needed to investigate these in terms of their cytokine and transcription factor signature and whether the balance between different T cell subsets for this antigen is stable. In previous work, McSorley and colleagues showed that the Th cell response to an individual antigen could differ depending upon when after infection the response was assessed (40). Moreover, ESAT-6 can inhibit IFN-γ production by T cells in response to M. tuberculosis, despite Th1 responses to ESAT-6 being detected in humans and mice *in vivo* (45, 46). Therefore, in our model, it is possible that secretion from Salmonella enables ESAT-6 to suppress IFN-γ production, while ESAT-6 immobilized on the cell surface is unable to impart this function. Such a modulation of Th1 responses to *S*. Typhimurium has been observed in mice coimmunized with *S*. Typhimurium and soluble FliC (44). Furthermore, since individual antigens expressed by *S*. Typhimurium can contain multiple epitopes (47), presentation of ESAT-6 as a secreted, soluble antigen rather than restricted on the bacterial cell surface may alter the availability of epitopes presented by antigen-presenting cells, thereby influencing the induction and phenotype of responsive T cells.

Mice immunized with SL3261/sec generated a significantly higher and sustained antibody response than that of mice immunized with SL3261/surf. At 21 days postinfection, total ESAT-6-specific IgG levels were nearly 100-fold higher in the SL3261/sec-immunized mice than in SL3261/surf-immunized mice, yet from day 35 the antibody levels in both groups were comparable. This suggests that secretion of antigen by the RASV provides an early advantage in driving specific antibody responses. This may be useful if an early antibody response is necessary. The reasons for the discordant induction of early antibody responses to ESAT-6 seen after immunization with the different vectors may reflect the diverse B cell epitopes exposed on the secreted protein compared to those on surface-localized ESAT-6. Furthermore, structures that protrude from the cell surface, such as the LPS O chain, can restrict the access of immunoglobulin to the bacterial outer membrane (48, 49). Hence, when ESAT-6 is expressed on the bacterial cell surface, certain epitopes may be shielded by cell surface structures, while secreted ESAT-6 may be more accessible to B cells and antibody, thereby triggering a more rapid antibody response. Otherwise, the difference in IgG titers between surface and secreted antigens at day 21 may reflect the atypical antibody response to Salmonella induced in mice, which differs substantially from that observed to most soluble antigens (50). Thus, delivery of secreted antigen by an RASV strain can induce responses with kinetics that resemble those observed after immunization with a purified antigen in subunit vaccines yet offers the advantage of antigen delivery without the need for costly and time-consuming protein purification.

There are limitations to this study. It is not clear how the levels of antigen produced *in vitro* reflect those *in vivo* or if there were effects of ESAT-6 production on the biology of the host strain. Furthermore, although both surface-expressing and secreting strains induced robust immune responses, we do not know if the half-life of ESAT-6 differed depending upon if it was surface localized or secreted. Moreover, further mechanistic studies are required to determine why secreted antigen drives a rapid antibody response yet leads to a less pronounced Th1 response than that with surface-localized antigen. Also, given that induction of both T and B cell responses to RASVs is desirable for their maximum efficacy (12), it would be of great interest to develop a functional assessment of the response, for instance, to determine whether our observed ESAT-6-specific T and B cell immune responses toward cell surface or secreted ESAT-6 confer protection against challenge with M. tuberculosis. In summary, this report provides a detailed analysis of immune responses toward differentially presented antigen and shows that altering the site of antigen localization provides a flexible platform for modulating the magnitude and timing of T and B cell responses to the target antigen. This is attractive for the design of RASVs but is also of interest to further our understanding of how antigen-specific responses to bacterially expressed antigens develop.

## MATERIALS AND METHODS

### Bacterial strains and growth conditions.

Escherichia coli NEB 5-alpha (high-efficiency competent cells; NEB) and TOP10 (Invitrogen) were used for cloning. E. coli TOP10 and BL21*(DE3) (Novagen) were used in secretion experiments and for large-scale protein expression. Salmonella enterica Typhimurium SL3261 (51) was used in mouse immunization studies. Bacteria were grown at 37°C in Luria-Bertani broth (LB) or on LB agar (1.5% [wt/vol]) plates supplemented with 100 μg/ml carbenicillin, as appropriate.

### Preparation of electrocompetent Salmonella cells and electroporation.

An overnight culture of *S*. Typhimurium SL3261 was inoculated into fresh LB medium and grown at 37°C with aeration to an optical density at 600 nm (OD_600_) of ∼0.8. The culture was incubated in a 42°C shaker for 15 min, followed by 10 min of incubation on ice (with shaking). The cells were harvested by centrifugation, washed twice in ice-cold MOPS buffer (20% glycerol, 1 mM MOPS [morpholinepropanesulfonic acid]), and resuspended in MOPS buffer. Cells were then either used immediately or snap-frozen and stored at −80°C for later use. For electroporation, cells were mixed with plasmid DNA and transferred immediately into a cold 0.2-cm cuvette. The cells were pulsed at 2.5 kV in an electroporator and immediately supplemented with warm SOC (New England Biolabs) medium. The cells were recovered for 1.5 h at 37°C with aeration and plated onto appropriate selection agar plates.

### Growth curves.

Overnight cultures were diluted in fresh LB broth (with carbenicillin where appropriate) to an OD_600_ of 0.05 and incubated at 37°C for 7 h with aeration. The culture OD_600_ was recorded hourly for ∼7 h. Three replicates of each culture were used to generate growth curves.

### Confirmation of protein secretion in E. coli and *S*. Typhimurium.

Overnight bacterial cultures were diluted in fresh LB medium containing carbenicillin and grown at 37°C with aeration. Cultures containing pettac vector derivatives were grown without induction, and culture supernatants were collected at 5 h postinoculation. Cultures containing derivatives of pASK or pET vectors were grown to an OD_600_ of ∼0.5, followed by induction of protein expression with anhydrotetracycline (200 μg/liter) or IPTG (isopropyl-β-d-thiogalactopyranoside) (0.5 mM), respectively. The supernatants for secreted protein analysis were collected at 1.5 to 2 h postinduction. Secreted protein and cell envelope fractions were prepared as described previously (18) and analyzed by SDS-PAGE and Western immunoblotting alongside the whole-cell lysates.

### Molecular biology techniques, SDS-PAGE, Western immunoblotting, indirect flow cytometry, and immunofluorescence microscopy.

All techniques were performed as described previously (18). Briefly, proteins were separated in NuPAGE 4 to 12% bis-Tris/MES gels (Life Technologies). Protein transfer to nitrocellulose membranes was done using an iBlot dry blot system (Life Technologies) per the manufacturer's instructions. For ESAT-6 detection by Western immunoblotting, primary monoclonal anti-ESAT-6 (Abcam) (1/2,000 dilution) and secondary alkaline phosphatase (AP)-conjugated anti-mouse (Sigma) antibodies were used. Measurement of band intensities was done using Quantity One software (with a rolling disk of 100 for lane background subtraction applied). The peak density obtained for each detected ESAT-6 band was multiplied by a dilution factor to generate the relative level of ESAT-6. For immunofluorescence microscopy and flow cytometry, polyclonal rabbit anti-ESAT-6 (1/300 dilution) and Alexa Fluor 488-conjugated goat anti-rabbit (1/500 dilution) (Invitrogen) antibodies were used as primary and secondary antibodies, respectively. When necessary, the cells were fixed and permeabilized. For this purpose, cell pellets were incubated in 4% paraformaldehyde–phosphate-buffered saline (PBS) for 20 min on ice and washed thoroughly with PBS. The fixed cells were permeabilized in PBS-0.1% Triton X for 45 min at room temperature, washed twice with PBS, incubated with 100 μg/ml lysozyme and 5 mM EDTA for 45 min at room temperature, and again washed with PBS before labeling.

### Plasmid construction.

To generate ESAT-6-Pet expression cassettes in the pettac vector, an *esxA-pet* chimeric DNA was PCR amplified from pASK-ESAT6-Pet-BB and pASK-ESAT6-Pet* (Table 1) with the NdeI-Pet-F and XhoI-Pet-R primers, respectively (Table 2). The PCR fragments obtained were digested with NdeI and XhoI and ligated into the pettac backbone, excised from pettacOVA with the same restriction enzymes, generating pettac-ESAT6-Pet (SL3261/sec) and pettac-ESAT6-Pet* (SL3261/surf), respectively. To construct pettac-ESAT6-Pet-cyto, an *esxA-pet* fragment lacking the *pet* signal sequence was PCR amplified from pASK-ESAT6-Pet-BB by use of the NdeI-(noSS)-ESAT6-F and XhoI-Pet-R primers (Table 2), followed by ligation into NdeI- and XhoI-digested pettacOVA. To construct pASK-His_6_-ESAT6-Pet-BP, the fragment encoding ESAT-6 was PCR amplified from pASK-ESAT6-Pet-BB with primers BglII-ESAT6-F and PstI-ESAT6-R and cloned into BglII- and PstI-digested pASK-His_6_-Pet. pET-His_6_-ESAT6-Pet-BP was created by subcloning of the SacI-SalI His_6_-ESAT6-Pet fragment from pASK-His_6_-ESAT6-Pet-BP into pET-Pet digested with SacI and SalI. All final constructs were checked by appropriate restriction digestion and/or sequencing with relevant primers (Table 2) (18).

**TABLE 2:** Primers used in this study

Primer	Sequence (5′-3′)^*a*^
NdeI-pet-F	TTAA**CATATG**AACAAAATCTACTCTATCAAATAC
XhoI-pet-R	AACG**CTCGAG**TTATCAGAAAGAGTAACGGAAG
HindIII-pet-R	CG**AAGCTT**TTATCAATGATGATGATG
NdeI-(noSS)-ESAT6-F	TTAT**CATATG**TCTCTGAAAATCTCTCAGGCGG
BglII-ESAT6-F	GCG**AGATCT**GATGACCGAACAGCAGTGGAAC
PstI-ESAT6-R	ATC**CTGCAG**AGCCGCCAGCGAACATGC
pET22b_F_seq	GTGATGTCGGCGATATAGGC

a Restriction sites are shown in bold.

### Large-scale protein expression and purification.

A fresh overnight culture of BL21*(DE3)/pET-His_6_-ESAT-6-Pet-BP was diluted 1/100 in 2 liters of LB medium with carbenicillin and grown at 37°C with aeration to an OD_600_ of ∼0.5, followed by induction of protein expression with 0.5 mM IPTG. After 2 h, the cells were removed by centrifugation (6,000 × *g*, 30 min, 4°C), and the supernatant was filtered through a 0.22-μm filter and supplemented with a protease inhibitor cocktail (Roche). The supernatant was applied to a buffer-equilibrated 1-ml HisTrap column (GE Healthcare) at a low rate at 4°C by use of a peristaltic pump. The column was connected to an Akta purifier, and the standard protocol for His-tagged protein purification under native conditions was followed. The equilibration/wash buffer contained 50 mM sodium phosphate, 500 mM NaCl, and 10 mM imidazole (pH 8.0), while the elution buffer contained 50 mM sodium phosphate, 500 mM NaCl, and 500 mM imidazole (pH 8.0). The eluted His-tagged protein was buffer exchanged into 10 mM Tris, 30 mM NaCl, pH 8.0, concentrated through a 30-kDa Vivaspin centrifugation device (Sartorius), and quantified using a Bradford protein assay kit (Bio-Rad). The protein sample was aliquoted and stored at −20°C until further use.

### Mouse immunization studies.

Wild-type C57BL/6 mice were purchased from HO Harlan Olac Ltd. (Bicester, United Kingdom). The mice were maintained under standard animal housing conditions in accordance with local and UK Home Office regulations. Overnight cultures of Salmonella strains for immunization studies were inoculated into fresh LB medium (with carbenicillin where appropriate) at a 1/20 dilution and grown at 37°C to an OD_600_ of ∼1. The cells from 1 ml of culture were harvested by centrifugation and washed twice with PBS. The cells were resuspended in 1 ml PBS. Naive female C57BL/6 mice (6 to 8 weeks old) were injected intraperitoneally (i.p.) with 5 × 10^5^ CFU. The exact injected dose was confirmed by plating dilutions of the cell suspension used for immunization on LB agar plates without antibiotic. The mouse cohorts included 3 to 8 animals per construct. An untransformed Salmonella strain was used as a negative control. For *in vivo* restimulation of memory cells, mice were injected intravenously (i.v.) with 100 μg T-dependent ESAT-6 peptide (M. tuberculosis ESAT-6 4-17 [QQWNFAGIEAAASA]; Proimmune) in 100 μl PBS 4 h prior to sacrifice. The mice were sacrificed at 7 or 42 days postimmunization, and spleens, livers, and blood were retained for analysis. For longer experiments, blood samples were also taken from a superficial tail vein at 7, 21, and 35 days postimmunization. The blood samples were allowed to clot at 37°C for 1 h, and the serum was separated by centrifugation at 15,000 × *g* for 20 min. The serum was aliquoted and stored at −80°C. Each immunization experiment was repeated at least twice.

### Determining bacterial burdens in mouse organs.

Weighed liver sections and whole spleens were passed through a 70-μm nylon cell strainer (BD Falcon) with 1 to 3 ml of PBS. The collected cell suspensions were diluted in PBS and plated onto LB agar plates without selection. The recovered colonies were scored, and the bacterial burden per whole organ was calculated. One hundred randomly selected colonies (or all if fewer were recovered) from each mouse's spleen and liver were patch plated onto a pair of LB agar plates, one supplemented with carbenicillin, to determine plasmid retention. A few antibiotic-resistant colonies (one per mouse) were picked at random and tested for antigen secretion as described above.

### Analysis of T cell responses.

ESAT-6-specific T cell responses were analyzed 7 and 42 days after immunization. To isolate splenocytes, spleens were passed through a 70-μm nylon cell strainer and washed three times with RPMI medium. The cells were harvested by centrifugation at 300 × *g* for 6 min at 4°C, resuspended in 1 ml of red blood cell lysis buffer (Sigma), and incubated at room temperature for 5 min. RPMI medium (Gibco) was then added directly to the lysed solution, ensuring that the clumps were resuspended. The cells were harvested as described above, resuspended in 5 ml ice-cold staining buffer (SB; Dulbecco's PBS [DPBS], 2.5 mM EDTA, 10% fetal calf serum [FCS]), and passed through an additional 70-μm strainer. One third of the cell mixture was pelleted as described above and resuspended in 100 μl of SB containing a phycoerythrin (PE)-conjugated ESAT-6 tetramer at a 1/100 dilution [class II MHC I-A(b) M. tuberculosis ESAT-6; NIH Tetramer Core Facility] and brefeldin A (10 μg/ml). The reaction mixtures were incubated for 1 h in a room temperature water bath. The cells were washed twice with SB, pelleted as described above, and resuspended in 400 μl SB. Two hundred microliters of tetramer-stained cells was used for standard T/B cell receptor staining with a cocktail of mouse-specific fluorescent antibodies (BD Biosciences), while 50-μl aliquots of unstained splenocytes were used for single staining reactions. The reaction mixtures were incubated on ice in the dark for 0.5 to 1 h, washed twice with SB, and fixed and permeabilized with Cytofix/Cytoperm Plus (BD Biosciences) overnight at 4°C. The cells were washed as described above and stained for intracellular interleukin-2 (IL-2) (Alexa Fluor 488) and IFN-γ (PE-Cy7) (BD Biosciences) or IFN-γ only for 1 h on ice. Washed cells were supplemented with 10 μl of Spherotech blank particles for cell enumeration. Samples were run on a BD LSR Fortessa X-20 flow cytometer and analyzed using FlowJo software.

### ELISA.

A standard indirect enzyme-linked immunosorbent assay (ELISA) protocol was used for detection of the antigen-specific IgG in the immunized mouse sera. Briefly, Nunc MaxiSorp 96-well plates were coated with 5 μg/ml of purified His_6_-ESAT-6-Pet-BP protein or *S*. Typhimurium LPS (Sigma) in 0.1 M carbonate buffer, pH 9.6 (overnight, 4°C). The plates were washed three times in wash buffer (PBS, 0.05% Tween 20), incubated with PBS-1% bovine serum albumin (BSA) for 1 h at 37°C, and washed as described above. The plates were incubated for 1 h at 37°C with 3-fold serial dilutions of mouse sera in dilution buffer (PBS, 0.05% Tween 20, 1% BSA), followed by three washes with wash buffer. The plates were incubated with a relevant AP-conjugated secondary antibody in dilution buffer for 1 h at 37°C (goat anti-mouse IgG–AP [1/10,000 dilution; Sigma] for protein- and LPS-specific total IgG or goat anti-mouse IgG1–AP, goat anti-mouse IgG2a–AP, or goat anti-mouse IgG2b–AP [1/2,000; Southern Biotech] for protein-specific IgG isotypes). The plates were washed as described above and developed using SigmaFAST *p*-nitrophenyl phosphate substrate (Sigma); 405-nm readings were taken at regular intervals by use of a Labsystems Multiscan MS plate reader. The IgG titers were estimated using the specified threshold OD method.

### Statistical analysis.

All statistical analyses were done using Prism software. The nonparametric Mann-Whitney test was used for pairwise comparisons (with statistical significance set at *P* values of ≤0.05).

### Study approval.

All animal procedures were carried out in strict accordance with local ethical approval from the University of Birmingham and the UK Home Office (project license 30/2850) as covered by the Animals (Scientific Procedures) Act 1986.

## Supplementary Material

Supplemental material
