# Supplementary material for: Antigen Localization Influences the Magnitude and Kinetics of Endogenous Adaptive Immune Response to Recombinant Salmonella Vaccines
Source: Infect Immun. 2017 Nov 17;85(12):e00593-17. doi: 10.1128/IAI.00593-17 (PMC5695123; doi:10.1128/IAI.00593-17)
Supplement: Supplemental material [file supp_85_12_e00593-17__index.html]

Supplemental material 

# Antigen Localization Influences the Magnitude and Kinetics of Endogenous Adaptive Immune Response to Recombinant Salmonella Vaccines

## Supplemental material

- Supplemental file 1 -

  Table S1. Analysis of vector stability in CFUs recovered 7 days postimmunization from spleens and livers of mice infected with ESAT-6-expressing RASVs. Fig. S1. Relative ESAT-6 levels in SL3261/cyto, Sl3261/sec, SL3261/surf, and SL2361/vec by quantitative Western blotting analysis.

  PDF, 407K
