# Supplementary material for: Antigen Localization Influences the Magnitude and Kinetics of Endogenous Adaptive Immune Response to Recombinant Salmonella Vaccines
Source: Infect Immun. 2017 Nov 17;85(12):e00593-17. doi: 10.1128/IAI.00593-17 (PMC5695123; doi:10.1128/IAI.00593-17)
Supplement: Supplemental material [file IAI.00593-17_zii999092210s1.pdf]

Supplementary Table 1. Analysis of vector stability in CFUs recovered 7 days post-immunisation from spleens and livers of mice infected with ESAT-6-expressing RASVs**.**

Construct	Mouse number	% Cb+ CFU from spleen	% Cb+ CFU from liver
**SL3261/sec**	1	100	94
	2	91	97
	3	100	100
	4	100	92
	5	99	94
**SL3261/surf**	1	100	92
	2	97	86
	3	86	100
	4	89	91
	5	85	95
**SL3261/cyto**	1	100	97
	2	78	95
	3	94	95

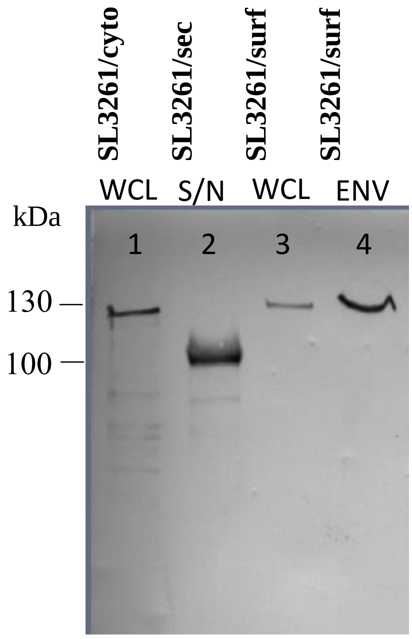

SL3261 strain/	Peak Relative Density	Peak Density x Dilution Factor
cyto	0.38	4.37
sec	0.50	4.98
surf (WCL)	0.15	4.00
surf (ENV)	0.44	4.36

Figure S1. Relative ESAT-6 levels in SL3261/cyto, Sl3261/sec, SL3261/surf and SL2361/vec by quantitative Western blotting analysis. For quantification, samples of cell cultures in late exponential phase were taken concomitantly with analysis of protein localisation presented in Figure 1. ESAT-6 protein was detected using monoclonal ESAT-6 antibodies as described in Materials and Methods. Measurement of band intensity was done using Quantity One software (rolling disk of 100 for lane background subtraction applied). The Peak density (table column 2) obtained for each detected ESAT-6 band was multiplied by a dilution factor to generate the relative levels of ESAT-6 (table column 3). Lanes 1 and 3 - whole cell lysate (WCL); lane 2 - TCA precipitated culture supernatant (S/N) and lane 4 – cell envelope fraction (ENV).
